# Supplementary material for: Enhancing tensile strength of 3D-printed wood-PLA composites via a particle swarm optimization framework
Source: PLoS One. 2026 Mar 24;21(3):e0344945. doi: 10.1371/journal.pone.0344945 (PMC13012462; doi:10.1371/journal.pone.0344945)
Supplement: Attachment — Submitted filename: report.pdf [file pone.0344945.s001.pdf]

The manuscript presents a well-structured and technically sound study that successfully integrates the Taguchi method, ANOVA, FEA, and PSO to optimize the tensile strength of 3D-printed Wood-PLA composites. The research is relevant and addresses a clear gap in the literature concerning the application of intelligent optimization techniques in sustainable additive manufacturing. The experimental design is robust, and the results are promising, showing a significant 28% improvement in tensile strength.

However, the manuscript requires revisions to enhance its academic rigor, clarity, and impact. Key areas for improvement include strengthening the introduction and discussion sections, justifying methodological choices, improving data presentation, and refining the English language and flow. The specific, actionable comments below are intended to help the authors improve the manuscript for publication.

1. Title

- **Evaluation:** The title is descriptive but slightly long and starts with "To develop," which is not standard for a research article title.
- **Suggestions:**
  - Shorten and rephrase to be more concise and impactful.
  - **Proposed Title:** "Enhancing Tensile Strength of 3D-Printed Wood-PLA Composites via a Particle Swarm Optimization Framework"

2. Abstract

- **Evaluation:** The abstract covers the essential elements but could be more focused on the present study's findings. The mention of "previous studies" is unnecessary here. The numerical results are the highlight and should be presented more clearly.
- **Suggestions:**
  - Remove the sentence on previous studies lacking intelligent optimization; this belongs in the introduction.
  - Start directly with the objective of this work.
  - Explicitly state the key optimized parameters and the resulting tensile strength earlier.
  - Ensure the 28% improvement is clearly linked to the baseline (e.g., "compared to the average strength of non-optimized configurations").
  - Grammar and phrasing need polishing for conciseness.

3. Introduction

- **Evaluation:** This is the weakest section of the manuscript. It provides a basic background but lacks depth and a compelling narrative. The literature review is insufficient, especially regarding recent (post-2023) studies and the specific application of PSO in polymer/composite 3D printing. The research gap and the novelty of the current work are not explicitly and powerfully stated.
- **Suggestions:**
  - **Structure:** Re-structure to follow: (1) Importance of FDM and bio-composites (Wood-PLA), (2) Challenge of parameter optimization, (3) Review of existing methods (Taguchi, RSM) and their limitations, (4) Introduction of nature-inspired algorithms (like PSO) and their *potential* in AM, noting the scarcity of their application to Wood-PLA, (5) Clear statement of the research gap, (6) Explicitly state the novelty and contribution of this work (e.g., first integrated Taguchi-ANOVA-FEA-PSO framework for Wood-PLA tensile strength).
  - **Literature:** Incorporate the suggested and other recent references (2023-2024). For example:
    - Discuss studies on PLA/wood dust optimization using Grey Relational Analysis (https://doi.org/10.1177/09544089241241460) to contrast with your computational approach.

        - Cite works on PSO in other AM contexts to justify its selection (e.g., https://doi.org/10.1016/j.measurement.2025.117405).
        - Include other relevant composite studies to show the broader context (e.g., https://doi.org/10.1002/pol.20250752).
    - **Novelty:** Clearly articulate how your study bridges the gap. For instance: "While statistical methods are common, this study introduces a robust PSO-based framework to achieve global optimization, validated by both experimental and numerical simulation, for sustainable Wood-PLA composites."

4. Materials & Methods

- **Evaluation:** Generally well-described, but several details and justifications are missing.
- **2.1 Materials:**
    - The description of the filament extruder is good, but the printer (Pratham 3.0) is not a widely known model. A key reference or a more detailed specification table would be beneficial.
- **2.2 Specimen Design and Fabrication:**
    - **Parameter Selection:** The authors must justify why these specific parameters (layer thickness, infill density, nozzle temperature) and their levels were chosen. Reference preliminary studies, material datasheets, or other literature (e.g., https://doi.org/10.1002/pol.20250752).
    - **Taguchi Method:** A citation for the Taguchi method and the L9 array is required.
- **3.4 Particle Swarm Optimization:**
    - **Presentation:** The current step-by-step list is clear but disrupts the narrative flow. It should be converted into a descriptive paragraph explaining the logic and process of PSO.
    - **Flowchart:** It is **highly recommended** to add a flowchart illustrating the overall methodology of the study, integrating the Taguchi DOE, experimental testing, ANOVA, PSO optimization, and FEA validation. A specific flowchart for the PSO algorithm (as in https://doi.org/10.1016/j.measurement.2025.117405) would significantly improve clarity.
    - **Algorithm Choice:** The authors should briefly justify why PSO was chosen over other optimization algorithms (e.g., Genetic Algorithm, Simulated Annealing). Mentioning its simplicity, convergence speed, and successful applications in similar engineering problems would suffice.

5. Results & Discussion

- **Evaluation:** The results are presented comprehensively with tables and graphs. However, the discussion is largely descriptive and lacks critical depth. It needs to interpret the results in the context of the existing literature and explain the underlying physical mechanisms.
- **Suggestions:**
    - **4.1 & 4.2 (Tensile Strength Analysis):** The text describes "what" happened but not deeply "why." For instance, explain the micro-mechanical reasons behind better interlayer adhesion with thinner layers and higher temperatures, linking it to the SEM results (Figure 11).
    - **4.2.4 Influence of Infill Pattern:** The discussion on the Cuboid pattern's performance is good. The contradictory finding for the Triangular pattern at 0.3 mm layer thickness needs a more robust explanation. Is it due to a specific interaction effect? This should be explored using the ANOVA interaction plots or hypothesized more clearly.
    - **4.5 PSO Results:** The convergence behavior of the PSO algorithm should be shown (e.g., a plot of fitness value vs. iteration). This is crucial to demonstrate the algorithm's efficiency and stability.

- **4.6 Confirmation Test (ANOVA):** Table 4 appears incorrect. The Degrees of Freedom (df) for a factor with 3 levels should be 2, not 1. The Sum of Squares (SS) values seem illustrative rather than calculated. The authors must provide the *actual* ANOVA results from their data analysis. The percentage contribution is a valuable metric and should be recalculated correctly.
- **Strengthen Discussion:** Compare your optimal tensile strength (46.41 MPa) with values reported in the literature (e.g., the studies mentioned for the introduction). Discuss why your value is higher or lower. Elaborate on how the PSO framework provided an advantage over using only the Taguchi method.

6. Conclusion

- **Evaluation:** The conclusion is currently a list of bullet points. This is not standard for a scientific paper and lacks a narrative that synthesizes the findings.
- **Suggestions:**
  - Rewrite into one or two coherent paragraphs.
  - Summarize the main findings and the most optimal parameter set.
  - Reiterate the core contribution (the successful development and validation of the PSO framework).
  - Mention the practical implications of the work for designing high-strength, sustainable 3D-printed parts.
  - Provide specific and forward-looking recommendations for future work (e.g., multi-objective optimization of strength, cost, and print time; application to other biocomposites; investigation of dynamic mechanical properties).

7. Figures and Tables

- **Evaluation:** The data is well-organized, but the quality and integration can be improved.
- **Suggestions:**
  - **Image Quality:** Ensure all figures (especially SEM images in Figure 11) are of high resolution and clearly labeled.
  - **Figure References:** All figures mentioned in the text (Figures 1, 2, 3, 4, 5, 6, 7, 8, 9, 10, 11, 12, 13, 14) must be included in the submitted manuscript file. Their captions should be descriptive enough to understand without reading the main text.
  - **Table 4 (ANOVA):** As mentioned, this table must be corrected with proper statistical analysis.
  - **New Figure:** Strongly recommend adding a methodology flowchart and a PSO convergence plot.

8. Data Availability Statement

- **Evaluation:** The current statement "The data will be available on request from the corresponding author" is **not sufficient** for PLOS ONE. The journal requires data to be in a public repository or included as Supporting Information.
- **Suggestion:** Change the statement to: "All relevant data are within the manuscript and its Supporting Information files." Alternatively, deposit the raw data (Taguchi L9 results, tensile test data) in a public repository like Figshare or Mendeley Data and provide the DOI.

9. Language and Grammar

- **Evaluation:** The manuscript requires thorough proofreading to correct grammatical errors, improve sentence structure, and enhance clarity.
- **Examples:**
  - "There are numerous AM methods that Fused Deposition Modelling (FDM) is one of the most popular..." -> "Among numerous AM methods, Fused Deposition Modelling (FDM) is one of the most popular..."
  - "Mechanical properties of FDM produced parts and the tests undertaken on aspects of tensile strength have been a prime area of study..." -> "The mechanical properties of

FDM-produced parts, particularly tensile strength, have been a primary research focus..."
    - "The improvement represents nearly 28 % higher strength compared to non-optimized samples" -> "This optimized configuration resulted in a tensile strength nearly 28% higher than the average of non-optimized samples."
- **Suggestion:** Consider using professional editing services or a native English-speaking colleague for a final review.

Summary of Major Revisions Required:

1. **Restructure and Strengthen the Introduction:** Clearly define the research gap, incorporate recent literature, and explicitly state the novelty and contributions of the work.
2. **Improve the Discussion:** Move beyond description to provide a critical interpretation of results, compare with literature, and explain underlying mechanisms. Justify the choice of PSO and show its convergence.
3. **Correct and Enhance Methodology:** Justify the selection of parameters and their levels. Correct the ANOVA table (Table 4). Integrate the PSO steps into a flowing text and add a methodology/PSO flowchart.
4. **Revise Presentation:** Change the conclusion to paragraph format. Ensure all figures are present and of high quality. Fulfill PLOS ONE's data availability policy.
5. **Thoroughly Proofread:** Correct grammatical errors and improve language fluency throughout the manuscript.

The study has strong potential, and addressing these points will significantly enhance its quality, clarity, and impact. I look forward to reviewing a revised version.
