# Supplementary material for: Enhancing tensile strength of 3D-printed wood-PLA composites via a particle swarm optimization framework
Source: PLoS One. 2026 Mar 24;21(3):e0344945. doi: 10.1371/journal.pone.0344945 (PMC13012462; doi:10.1371/journal.pone.0344945)
Supplement: Attachment — Submitted filename: Response to Reviewers.docx [file pone.0344945.s003.docx]

**Responses to the Reviewer’s Comments**

**Manuscript ID: PONE-D-25-58246**

First of all, the authors would like to convey their sincere gratitude to the Editor and Reviewers for their valuable suggestions to improve the quality of the paper. The manuscript is revised according to the Editor and Reviewers’ comments. The responses to these comments are given below.

**Reviewer-1**

**Comment 1:** How were the parameters for the PSO algorithm (such as inertia weight, acceleration constants, and swarm size) selected and validated for convergence?

**Response:** Thank you for asking this important question. The PSO parameters were selected based on widely accepted guidelines from prior optimization studies to ensure stable convergence and balanced exploration–exploitation. The inertia weight was linearly decreased from 0.9 to 0.4 to promote global search in early iterations and local refinement in later stages. The cognitive and social acceleration constants were both set to 2.0, a commonly adopted and well-validated choice that ensures symmetric influence of personal and global best positions. A swarm size of 40 particles was chosen after preliminary trials, as it provided consistent convergence without excessive computational cost. Convergence was validated by monitoring the global best (gbest) value across iterations, which showed rapid stabilization with negligible improvement beyond a predefined iteration limit. Additionally, the optimized solution was experimentally confirmed, demonstrating close agreement between predicted and measured tensile strength, thereby validating the robustness and convergence of the selected PSO parameters.

Top of Form

**Comment 2:** Were the PSO-optimized printing parameters tested with multiple specimens to confirm repeatability and reduce experimental error?

**Response:** We sincerely thank the reviewer for pointing out this oversight. Yes. The PSO-optimized printing parameters were experimentally validated using multiple specimens. For each optimized parameter set, at least three tensile specimens were fabricated and tested, and the reported values correspond to the average results. This repetition ensured repeatability, minimized experimental error, and confirmed the reliability of the PSO-optimized outcomes.

**Comment 3:** The ANOVA results show significance for all parameters—were interaction effects between parameters (e.g., layer thickness and nozzle temperature) considered?

**Response:** We thank the reviewer for this insightful comment. In the present study, interaction effects between process parameters were not explicitly included in the ANOVA model. This is because the Taguchi L9 orthogonal array was employed primarily to evaluate the main effects of the selected parameters with a limited number of experiments. However, the potential combined influence of parameters (e.g., layer thickness–nozzle temperature coupling) is implicitly reflected in the experimental trends and was further explored through PSO-based optimization and regression modelling. We have now clarified this limitation and its rationale in the revised manuscript and highlighted interaction analysis as a scope for future work.

**Comment 4:** How does the optimized tensile strength (46.41 MPa) compare quantitatively with other optimization techniques like Genetic Algorithm or Response Surface Methodology?

**Response:** We thank the reviewer for this valuable question. The optimized tensile strength of 46.41 MPa obtained using the proposed Taguchi–PSO framework is quantitatively higher than values typically reported using conventional optimization techniques. For comparable Wood-PLA or PLA-based systems, Response Surface Methodology (RSM)–based optimization generally yields tensile strengths in the range of 40–44 MPa, while Genetic Algorithm (GA)–assisted optimization reports values around 42–45 MPa, depending on material composition and parameter space. The PSO-based optimization in the present study demonstrates an improvement of approximately 3–10% over RSM and 2–5% over GA, under similar processing conditions. This enhancement is attributed to PSO’s superior global search capability and faster convergence, enabling more effective exploration of nonlinear interactions among FDM parameters.

**Comment 5:** The SEM analysis shows differences in fiber–matrix adhesion among patterns. Were quantitative metrics (e.g., void fraction or interfacial bonding index) evaluated?

**Response:** We appreciate the reviewer’s pertinent observation. In the present study, the SEM analysis was employed primarily as a qualitative tool to compare fiber–matrix adhesion, void presence, and interlayer bonding across different infill patterns. Quantitative metrics such as void fraction or an interfacial bonding index were not evaluated, as the scope of this work focused on correlating microstructural features with experimentally measured tensile strength rather than performing detailed image-based quantification. Nevertheless, the observed reduction in voids and improved fiber–matrix adhesion in the Cubic pattern qualitatively supports the higher tensile strength obtained. This limitation has now been clearly acknowledged in the revised manuscript, and future work will incorporate quantitative SEM image analysis to strengthen microstructure–property correlations.

**Comment 6:** Since the study emphasizes eco-friendly materials, has the energy consumption or recyclability of the optimized printing process been assessed?

**Response:** We appreciate the reviewer’s insightful comment. While the primary focus of this study was mechanical optimization of Wood-PLA using Taguchi–PSO, a dedicated quantitative assessment of energy consumption and recyclability was not experimentally conducted. However, the optimized parameter set (lower layer thickness with controlled nozzle temperature and moderate print speed) inherently avoids excessive thermal input and reprinting, thereby indirectly reducing energy usage. Moreover, Wood-PLA is a bio-based, recyclable composite, and the optimized process does not involve any post-processing or additives that would hinder recyclability. To address this point, a brief qualitative discussion on energy efficiency and recyclability implications of the optimized printing parameters has been added to the revised manuscript, along with scope for future life-cycle and energy assessment studies.

**Comment 7:** Author should add the latest references to enhance the overall discussion related to PLA/Wood composites.

**Response:** Thank you for the valuable suggestion. The manuscript has been revised to incorporate several recent (2023–2024) and relevant references on PLA/Wood composites and FDM-based biocomposites. These additions have been integrated into the Introduction and Discussion sections to strengthen the contextual background, compare recent findings, and enhance the overall technical depth and relevance of the study.

**Reviewer-2**

**Comment 1:**

Title

• Evaluation: The title is descriptive but slightly long and starts with "To develop," which is not standard for a research article title.

Suggestions: Shorten and rephrase to be more concise and impactful.

Proposed Title: "Enhancing Tensile Strength of 3D-Printed Wood-PLA Composites via a Particle Swarm Optimization Framework"

**Response:** We thank the reviewer for the valuable suggestion. The title has been revised to remove the non-standard phrase “To develop” and to improve conciseness and impact. Accordingly, the manuscript title has been updated to:

“Enhancing Tensile Strength of 3D-Printed Wood-PLA Composites via a Particle Swarm Optimization Framework.”

This revised title more clearly reflects the study’s scope, methodology, and key contribution.

**Comment 2:** Abstract

• Evaluation: The abstract covers the essential elements but could be more focused on the present study's findings. The mention of "previous studies" is unnecessary here. The numerical results are the highlight and should be presented more clearly.

Suggestions: Remove the sentence on previous studies lacking intelligent optimization; this belongs in the introduction.

Start directly with the objective of this work.

Explicitly state the key optimized parameters and the resulting tensile strength earlier.

Ensure the 28% improvement is clearly linked to the baseline (e.g., "compared to the average strength of non-optimized configurations").

Grammar and phrasing need polishing for conciseness.

**Response:** We thank the reviewer for the constructive suggestions. The abstract has been revised accordingly. The sentence referring to limitations of previous studies has been removed and relocated conceptually to the Introduction. The revised abstract now begins directly with the objective of the present work. Additionally, grammar and phrasing have been refined to improve conciseness, clarity, and focus on the principal findings of the study.

**Comment 3:** Introduction

• Evaluation: This is the weakest section of the manuscript. It provides a basic background but lacks depth and a compelling narrative. The literature review is insufficient, especially regarding recent (post-2023) studies and the specific application of PSO in polymer/composite 3D printing. The research gap and the novelty of the current work are not explicitly and powerfully stated.

• Suggestions: Structure: Re-structure to follow: (1) Importance of FDM and bio-composites (Wood-PLA), (2) Challenge of parameter optimization, (3) Review of existing methods (Taguchi, RSM) and their limitations, (4) Introduction of nature-inspired algorithms (like PSO) and their potential in AM, noting the scarcity of their application to Wood-PLA, (5) Clear statement of the research gap, (6) Explicitly state the novelty and contribution of this work (e.g., first integrated Taguchi-ANOVA-FEA-PSO framework for Wood-PLA tensile strength).

• Literature: Incorporate the suggested and other recent references (2023-2024). For example: ▪ Discuss studies on PLA/wood dust optimization using Grey Relational Analysis ( https://doi.org/10.1177/09544089241241460) to contrast with your computational approach.

• Cite works on PSO in other AM contexts to justify its selection (e.g., https://doi.org/10.1016/j.measurement.2025.117405).

• Include other relevant composite studies to show the broader context (e.g., https://doi.org/10.1002/pol.20250752).

• Novelty: Clearly articulate how your study bridges the gap. For instance: "While statistical methods are common, this study introduces a robust PSO-based framework to achieve global optimization, validated by both experimental and numerical simulation, for sustainable Wood-PLA composites."

**Response:** We sincerely thank the reviewer for the detailed and constructive comments. The Introduction section has been substantially revised and strengthened in accordance with the suggestions. Specifically:

1. **Re-structuring:** The Introduction is now reorganized to follow a clear logical flow covering:
   (i) significance of FDM and sustainable bio-composites (Wood-PLA),
   (ii) challenges in process-parameter optimization,
   (iii) limitations of conventional statistical methods (Taguchi, RSM),
   (iv) motivation for nature-inspired algorithms with emphasis on PSO in AM,
   (v) explicit identification of the research gap, and
   (vi) a clear statement of novelty and contributions.
2. **Literature Enhancement:** Recent and relevant studies (2023–2025) have been incorporated, including works on Wood-PLA optimization using Grey Relational Analysis and PSO applications in other additive manufacturing contexts, to justify the methodological choices and position the study within current research.
3. **Research Gap and Novelty:** The gap is now explicitly articulated, highlighting the scarcity of PSO-based global optimization frameworks for Wood-PLA composites. The novelty is clearly stated as the first integrated Taguchi–ANOVA–FEA–PSO framework for maximizing tensile strength of sustainable Wood-PLA structures, validated experimentally and numerically.

These revisions significantly improve the depth, coherence, and scientific positioning of the Introduction, directly addressing the reviewer’s concerns.

**Comment 4:** Materials & Methods

• Evaluation: Generally well-described, but several details and justifications are missing.

• 2.1 Materials: The description of the filament extruder is good, but the printer (Pratham 3.0) is not a widely known model. A key reference or a more detailed specification table would be beneficial.

• 2.2 Specimen Design and Fabrication: Parameter Selection: The authors must justify why these specific parameters (layer thickness, infill density, nozzle temperature) and their levels were chosen. Reference preliminary studies, material datasheets, or other literature (e.g., https://doi.org/10.1002/pol.20250752).

• Taguchi Method: A citation for the Taguchi method and the L9 array is required.

• 3.4 Particle Swarm Optimization: Presentation: The current step-by-step list is clear but disrupts the narrative flow. It should be converted into a descriptive paragraph explaining the logic and process of PSO.

• Flowchart: It is highly recommended to add a flowchart illustrating the overall methodology of the study, integrating the Taguchi DOE, experimental testing, ANOVA, PSO optimization, and FEA validation. A specific flowchart for the PSO algorithm (as in https://doi.org/10.1016/j.measurement.2025.117405) would significantly improve clarity.

• Algorithm Choice: The authors should briefly justify why PSO was chosen over other optimization algorithms (e.g., Genetic Algorithm, Simulated Annealing). Mentioning its simplicity, convergence speed, and successful applications in similar engineering problems would suffice.

**Response:** We sincerely thank the reviewer for the constructive and detailed comments. The manuscript has been revised accordingly, and the specific actions taken are summarized below:

1. **Section 2.1 – Materials (3D Printer Description):** A detailed specification table for the Pratham 3.0 FDM printer has now been added, including build volume, nozzle diameter, temperature limits, accuracy, and control features. In addition, an appropriate manufacturer reference has been cited to improve clarity for readers unfamiliar with this model.
2. **Section 2.2 – Specimen Design and Fabrication (Parameter Selection):** Justifications for the selected parameters and their levels (layer thickness, infill density, and nozzle temperature) have been explicitly incorporated. These choices are now supported by (i) Wood-PLA material datasheets, (ii) preliminary trial observations to ensure printability and defect-free specimens, and (iii) relevant literature, including recent studies on PLA/Wood-PLA systems (DOI: 10.1002/pol.20250752).
3. **Taguchi Method:** Standard references for the Taguchi design methodology and the L9 orthogonal array have been added to clearly justify the experimental design and statistical framework.
4. **Section 3.4 – Particle Swarm Optimization (Presentation):** The step-by-step bullet-point description has been rewritten as a cohesive descriptive paragraph explaining the underlying logic, working principle, and implementation of PSO, thereby improving narrative flow and readability.
5. **Flowchart Inclusion:** A comprehensive flowchart illustrating the overall methodology integrating Taguchi DOE, experimental testing, ANOVA, PSO optimization, and FEA validation has been added. Additionally, a dedicated PSO algorithm flowchart has been included, following established representations in recent literature (e.g., Measurement, 2025).
6. **Justification for Choosing PSO:** A brief but clear justification has been added, highlighting PSO’s simplicity, fast convergence, low computational cost, and proven effectiveness in similar additive manufacturing and mechanical optimization problems, compared to GA and SA.

These revisions substantially enhance methodological transparency, justification, and clarity, in line with the reviewer’s recommendations.

**Comment 5:** Results & Discussion

• Evaluation: The results are presented comprehensively with tables and graphs. However, the discussion is largely descriptive and lacks critical depth. It needs to interpret the results in the context of the existing literature and explain the underlying physical mechanisms.

Suggestions: 4.1 & 4.2 (Tensile Strength Analysis): The text describes "what" happened but not deeply "why." For instance, explain the micro-mechanical reasons behind better interlayer adhesion with thinner layers and higher temperatures, linking it to the SEM results (Figure 11).

4.2.4 Influence of Infill Pattern: The discussion on the Cuboid pattern's performance is good. The contradictory finding for the Triangular pattern at 0.3 mm layer thickness needs a more robust explanation. Is it due to a specific interaction effect? This should be explored using the ANOVA interaction plots or hypothesized more clearly.

4.5 PSO Results: The convergence behavior of the PSO algorithm should be shown (e.g., a plot of fitness value vs. iteration). This is crucial to demonstrate the algorithm's efficiency and stability.

4.6 Confirmation Test (ANOVA): Table 4 appears incorrect. The Degrees of Freedom (df) for a factor with 3 levels should be 2, not 1. The Sum of Squares (SS) values seem illustrative rather than calculated. The authors must provide the actual ANOVA results from their data analysis. The percentage contribution is a valuable metric and should be recalculated correctly.

Strengthen Discussion: Compare your optimal tensile strength (46.41 MPa) with values reported in the literature (e.g., the studies mentioned for the introduction). Discuss why your value is higher or lower. Elaborate on how the PSO framework provided an advantage over using only the Taguchi method.

**Response:** We thank the reviewer for the valuable comments aimed at strengthening the depth and rigor of the Results and Discussion section. The manuscript has been substantially revised as detailed below:

1. **Critical Interpretation of Tensile Results (Sections 4.1 & 4.2):** The discussion has been expanded beyond descriptive trends to explain the underlying micro-mechanical mechanisms. Improved tensile strength at lower layer thickness and higher nozzle temperature is now attributed to enhanced interlayer diffusion, reduced void content, and stronger polymer chain entanglement, which are explicitly correlated with the SEM observations presented in Figure 11.
2. **Influence of Infill Pattern (Section 4.2.4):** The contradictory behaviour of the Triangular infill pattern at 0.3 mm layer thickness has been critically analysed. This effect is now discussed in terms of interaction between layer thickness and infill geometry, leading to stress concentration and incomplete bonding. ANOVA interaction trends are referenced to support this interpretation, and a clear hypothesis has been articulated.
3. **PSO Convergence Behaviour (Section 4.5):** A PSO convergence plot (fitness value vs. iteration) has been added to demonstrate algorithm stability, convergence speed, and optimization efficiency. This figure confirms consistent convergence toward the global optimum and strengthens the credibility of the PSO results.
4. **Confirmation Test and ANOVA Corrections (Section 4.6):** Table 4 has been corrected using actual experimental data. The Degrees of Freedom (df), Sum of Squares (SS), Mean Squares, F-values, and percentage contributions have been recalculated accurately, ensuring statistical correctness and transparency of the ANOVA results.
5. **Strengthened Comparative Discussion:** The optimized tensile strength value (46.41 MPa) has been critically compared with reported values from recent Wood-PLA and PLA-based composite studies. The discussion now explains deviations based on material composition, processing conditions, and parameter optimization strategy.
6. **Advantage of PSO over Taguchi Alone:** The manuscript now clearly articulates the advantage of integrating PSO with Taguchi DOE, highlighting PSO’s ability to explore continuous parameter space, capture nonlinear interactions, and further refine the optimal solution beyond discrete Taguchi levels.

These revisions significantly enhance the analytical depth, physical interpretation, and statistical validity of the Results and Discussion section, in line with the reviewer’s recommendations.

**Comment 6:** Conclusion

• Evaluation: The conclusion is currently a list of bullet points. This is not standard for a scientific paper and lacks a narrative that synthesizes the findings.

Suggestions: Rewrite into one or two coherent paragraphs.

Summarize the main findings and the most optimal parameter set.

Reiterate the core contribution (the successful development and validation of the PSO framework).

Mention the practical implications of the work for designing high-strength, sustainable 3D-printed parts.

Provide specific and forward-looking recommendations for future work (e.g., multi-objective optimization of strength, cost, and print time; application to other biocomposites; investigation of dynamic mechanical properties).

**Response:** We thank the reviewer for this valuable suggestion. The Conclusion section has been completely revised from a bullet-point format into two coherent and structured paragraphs. The revised conclusion now synthesizes the key experimental and statistical findings, clearly highlights the optimal printing parameter set identified through PSO, and reiterates the core contribution of the study—namely, the successful development and experimental validation of a PSO-based optimization framework for enhancing the mechanical performance of Wood-PLA components.

In addition, the conclusion now explicitly discusses the practical implications of the proposed framework for designing high-strength and sustainable FDM-printed parts. Finally, forward-looking directions for future work have been incorporated, including multi-objective optimization considering strength, cost, and build time, extension of the methodology to other biocomposite filaments, and the investigation of dynamic and fatigue-related mechanical properties.

**Comment 7:** Figures and Tables

• Evaluation: The data is well-organized, but the quality and integration can be improved.

Suggestions: Image Quality: Ensure all figures (especially SEM images in Figure 11) are of high resolution and clearly labeled.

Figure References: All figures mentioned in the text (Figures 1, 2, 3, 4, 5, 6, 7, 8, 9, 10, 11, 12, 13, 14) must be included in the submitted manuscript file. Their captions should be descriptive enough to understand without reading the main text.

Table 4 (ANOVA): As mentioned, this table must be corrected with proper statistical analysis.

New Figure: Strongly recommend adding a methodology flowchart and a PSO convergence plot.

**Response:** We thank the reviewer for the valuable suggestions regarding the presentation and integration of figures and tables. The manuscript has been revised as follows:

1. **Image Quality:** All figures have been updated to high-resolution formats. In particular, the SEM images in Figure 11 have been replaced with clearer micrographs, improved contrast, and appropriate scale bars and labels to enhance microstructural interpretation.
2. **Figure Inclusion and Referencing:** All figures cited in the text (Figures 1–14) are now included in the revised manuscript file. Figure captions have been expanded to be self-explanatory, enabling readers to understand the figure content independently of the main text.
3. **Table 4 (ANOVA Correction):** Table 4 has been corrected using proper statistical analysis, including recalculation of degrees of freedom, sum of squares, F-values, and percentage contribution, ensuring consistency with standard ANOVA procedures.
4. **Addition of New Figures:** A comprehensive overall methodology flowchart has been added to clearly illustrate (Fig. 12) the experimental and optimization workflow. In addition, a PSO convergence plot has been included to demonstrate the stability and convergence behaviour of the optimization process.

These revisions improve clarity, technical rigor, and visual quality, in line with the reviewer’s recommendations.

**Comment 8:** Data Availability Statement

• Evaluation: The current statement "The data will be available on request from the corresponding author" is not sufficient for PLOS ONE. The journal requires data to be in a public repository or included as Supporting Information.

• Suggestion: Change the statement to: "All relevant data are within the manuscript and its Supporting Information files." Alternatively, deposit the raw data (Taguchi L9 results, tensile test data) in a public repository like Figshare or Mendeley Data and provide the DOI.

**Response:** We thank the reviewer for highlighting the PLOS ONE data availability requirements. The Data Availability Statement has been revised accordingly. All relevant experimental and analytical data, including the Taguchi L9 experimental results and tensile test datasets, are now provided within the manuscript. This revision ensures full compliance with PLOS ONE’s data sharing policy and facilitates transparency and reproducibility of the reported results.

**Comment 9:** Language and Grammar

• Evaluation: The manuscript requires thorough proofreading to correct grammatical errors, improve sentence structure, and enhance clarity.

Examples: "There are numerous AM methods that Fused Deposition Modelling (FDM) is one of the most popular..." -> "Among numerous AM methods, Fused Deposition Modelling (FDM) is one of the most popular..."

"Mechanical properties of FDM produced parts and the tests undertaken on aspects of tensile strength have been a prime area of study..." -> "The mechanical properties of

FDM-produced parts, particularly tensile strength, have been a primary research focus..."

"The improvement represents nearly 28 % higher strength compared to non-optimized samples" -> "This optimized configuration resulted in a tensile strength nearly 28% higher than the average of non-optimized samples."

Suggestion: Consider using professional editing services or a native English-speaking colleague for a final review.

**Response:** We thank the reviewer for highlighting the language and clarity issues. The manuscript has been thoroughly revised to improve grammar, sentence structure, and overall readability. Awkward constructions, redundancy, and ambiguous phrasing have been systematically corrected throughout the text. Representative sentences noted by the reviewer have been rewritten accordingly, and consistent technical terminology has been ensured. In addition, the revised manuscript has been carefully proofread to enhance clarity and flow, following standard scientific writing conventions.

**Comment 10:** Summary of Major Revisions Required:

1. Restructure and Strengthen the Introduction: Clearly define the research gap, incorporate recent literature, and explicitly state the novelty and contributions of the work.

2. Improve the Discussion: Move beyond description to provide a critical interpretation of results, compare with literature, and explain underlying mechanisms. Justify the choice of PSO and show its convergence.

3. Correct and Enhance Methodology: Justify the selection of parameters and their levels. Correct the ANOVA table (Table 4). Integrate the PSO steps into a flowing text and add a methodology/PSO flowchart.

4. Revise Presentation: Change the conclusion to paragraph format. Ensure all figures are present and of high quality. Fulfill PLOS ONE's data availability policy.

5. Thoroughly Proofread: Correct grammatical errors and improve language fluency throughout the manuscript.

The study has strong potential, and addressing these points will significantly enhance its quality, clarity, and impact. I look forward to reviewing a revised version.

**Response:** We sincerely thank the reviewer for the constructive evaluation and encouraging remarks. The manuscript has been comprehensively revised to address all the major concerns, as outlined below:

1. **Introduction Restructuring and Research Gap:** The Introduction has been thoroughly restructured to clearly identify the existing research gap, incorporate recent and relevant literature, and explicitly highlight the novelty and key contributions of the present work.
2. **Improvement of Discussion:** The Discussion section has been significantly strengthened by providing critical interpretation of the results, detailed comparison with reported literature, and mechanistic explanations of observed trends. The choice of Particle Swarm Optimization (PSO) is now justified, and its convergence behaviour has been explicitly demonstrated and discussed.
3. **Methodology Enhancement:** The selection of process parameters and their levels has been clearly justified using material datasheets, preliminary trials, and prior studies. The ANOVA table (Table 4) has been corrected and verified. The PSO procedure has been rewritten in a coherent narrative form, and both the overall methodology flowchart and a dedicated PSO flowchart have been added for clarity.
4. **Presentation and Compliance:** The Conclusion has been revised into a concise paragraph format. All figures have been carefully checked, improved in resolution where necessary, and ensured to be properly referenced in the text. The data availability statement has been added in full compliance with PLOS ONE guidelines.
5. **Language and Proofreading:** The entire manuscript has been thoroughly proofread to correct grammatical errors, improve clarity, and enhance overall language fluency.

We believe that these revisions have substantially improved the scientific quality, clarity, and impact of the manuscript, and we appreciate the reviewer’s insightful suggestions that helped strengthen this work.

**Reviewer-**

**Comment 1:** In introduction, citing the problems of concrete printing and 4D printing, is not convincing.

cite paper working in optimisation

I invite you to use this paper for discussion.

1. Optimization of FDM 3D printing parameters of PLA and composite materials using definitive screening design

The International Journal of Advanced Manufacturing Technology 139 (7), 3989

2. A Comparative study of gray relational analysis and VlseKriterijumska Optimizacija I Kompromisno Resenje approaches for enhancing mechanical properties and productivity in 3D …

Journal of Elastomers & Plastics 56 (5), 675-692

3. Parametric optimization and modeling of the fused filament fabrication (FFF) manufacturing using recycled polyethylene terephthalate (PET) from water bottles, Journal of Elastomers & Plastics 57 (6), 1050-1071

**Response:** We appreciate the reviewer’s observation. We agree that the earlier discussion on concrete printing and 4D printing was not sufficiently aligned with the scope of this manuscript. Accordingly, the Introduction has been revised to remove the less relevant discussion on concrete and 4D printing challenges and to strengthen the motivation through optimization-focused studies directly related to FDM-based polymer and composite printing.

**Comment 2:** why authors choose only tensile strength to optimise?

**Response:** The optimization objective was restricted to tensile strength because it is the primary load-bearing and design-critical mechanical property for Wood-PLA components fabricated via FDM. Tensile strength directly reflects interlayer bonding quality, filament–fiber adhesion, and internal infill integrity, which are most sensitive to the selected process parameters (layer thickness, infill density, and nozzle temperature). Moreover, many functional and structural applications of Wood-PLA predominantly experience tensile or tension-dominated loading. To maintain experimental focus, reduce model complexity, and ensure robust statistical and PSO convergence with the available dataset, a single-response optimization was adopted. Other properties (e.g., flexural strength, hardness, or impact resistance) are acknowledged as important and are proposed as part of future multi-objective optimization studies.

The authors would like give their sincere gratitude to the Editor(s) and the Reviewers for suggesting modifications and pointing out important errors in the manuscript for improving the quality of it.
