# Supplementary material for: Enhancing tensile strength of 3D-printed wood-PLA composites via a particle swarm optimization framework
Source: PLoS One. 2026 Mar 24;21(3):e0344945. doi: 10.1371/journal.pone.0344945 (PMC13012462; doi:10.1371/journal.pone.0344945)
Supplement: Attachment — Submitted filename: Response to Reviewers-2.docx [file pone.0344945.s004.docx]

**Responses to the Reviewer’s Comments**

**Manuscript ID: PONE-D-25-58246R1**

First of all, the authors would like to convey their sincere gratitude to the Editor and Reviewers for their valuable suggestions to improve the quality of the paper. The manuscript is revised according to the Editor and Reviewers’ comments. The responses to these comments are given below.

**EDITOR**

**Comment 1:** The references mentioned by the reviewers, is not mandatory.

**Response:** We respectfully acknowledge the editor’s observation. While the inclusion of the suggested references may not be mandatory, we have carefully evaluated them for technical relevance and contextual alignment with the present study. Wherever the cited works contributed to strengthening the theoretical background, optimization framework discussion, or recent advancements in additive manufacturing and PSO-based methodologies, they have been appropriately incorporated and critically discussed in the revised manuscript.

Top of Form

**Comment 2:** All highlights must be sentences. Correct the third one.

**Response:** We sincerely thank the editor for pointing out this oversight. We have converted the third highlight into a sentence.

**Comment 3:** All keywords must be found in the title or the abstract. Please recheck.

**Response:** We thank the editor for this insightful comment. We have checked and corrected accordingly.

**Comment 4:** The introduction must be maximum 2 pages.

**Response:** We sincerely thank the Editor for the valuable comment regarding the length of the Introduction section. In response, we have carefully revised and try to condensed the Introduction as much as possible.

While reducing the overall length, we retained essential background information and key literature citations that were specifically requested by the reviewers during the first revision. Redundant descriptions and repetitive discussions have been removed, and the content has been streamlined to focus on: (i) the research gap in PSO-based global optimization for Wood-PLA composites, (ii) the novelty of the integrated Taguchi–ANOVA–FEA–PSO framework, and (iii) the specific objective of maximizing tensile strength.

**Comment 5:** The novelty must be mentioned in the introduction, compared to the literature review.

**Response:** We thank the Editor for this valuable suggestion. The Introduction section has been revised to explicitly articulate the novelty of the present work in direct comparison with the existing literature. While prior studies predominantly employ statistical optimization methods such as Taguchi, GRA, ANN, and hybrid ML models for Wood-PLA composites, none report a fully integrated global optimization framework combining Taguchi–ANOVA–FEA–PSO specifically for tensile strength enhancement of sustainable Wood-PLA structures.

**Comment 6:** All process parameters need references, like 3600 rpm, 200 C, Table 2, etc.

**Response:** We thank the editor for this insightful comment. We have checked and corrected accordingly.

**Comment 7:** The scale bar must be added to Figures 3 and 4.

**Response:** Thank you for the valuable suggestion. We have checked and corrected accordingly.

**Comment 8:** Why Taguchi design? Write the reason in the main text. The regression is a linear curve-fitting and it is not proper for parameters with 3 levels.

**Response:** Thank you for the valuable comment. The following clarifications have been incorporated in the revised manuscript.

**(1) Justification for Taguchi Design**

The Taguchi L9 orthogonal array was selected because the study involves three independent control factors (layer thickness, infill density, and nozzle temperature), each at three discrete levels. A full factorial design would require 3^3^=27 experiments, whereas the L9 array reduces this to 9 trials while preserving balanced representation and orthogonality. This significantly minimizes experimental cost, material consumption, and machine time without compromising statistical robustness. Since the objective at this stage was screening and identification of dominant factors prior to metaheuristic optimization (PSO), Taguchi design provides an efficient and widely validated framework for parameter analysis in FDM-based composite studies. The rationale for selecting Taguchi design has now been explicitly added in Section 2.2.

**(2) Clarification Regarding Linear Regression and Three-Level Factors**

We agree that simple linear regression assumes a linear relationship and may not fully capture curvature effects inherent in three-level parameters. In the present study, regression equations were used primarily as empirical predictive models within the tested parameter range, not as higher-order response surface models. The three-level factors were treated as continuous variables within a restricted experimental domain, which allows first-order approximation. However, the global optimization and nonlinear interaction effects were addressed using Particle Swarm Optimization (PSO), which does not rely on linearity assumptions. A clarification has been added in Section 4.6 to explicitly state this limitation and the complementary role of PSO in overcoming linear modeling constraints.

**Comment 9:** What is the repeatability in Table 1?

**Response:** Thank you for the valuable question. Repeatability refers to the consistency of tensile strength measurements for identical parameter settings. For each L9 trial, three specimens were tested, and the average value was reported. The low standard deviation (σ = 2.99–3.57 MPa) confirms good experimental repeatability and measurement reliability.

**Comment 10:** More details of numerical simulations must be added, including the boundary condition, material properties, convergency of mesh, loading, etc.

**Response:** Thank you for the valuable suggestion. The revised manuscript now includes detailed numerical simulation parameters, including boundary conditions (fixed–displacement loading), material properties of Wood-PLA (elastic modulus, Poisson’s ratio), mesh type and refinement study for convergence, loading magnitude, and solver settings to ensure reproducibility and accuracy of FEA results.

**Comment 11:** Figure 5 is a result and it must be moved to the related part.

**Response:** Thank you for the valuable observation. Figure 5 presents FEA simulation results and is indeed part of the Results & Discussion section. Accordingly, it has been relocated from the methodology section to the appropriate Results section to maintain proper logical flow and structural consistency of the manuscript.

**Comment 12:** Figure 5 must be validated.

**Response:** Figure 5 has been validated by quantitatively comparing FEA-predicted tensile strength and stress distribution with experimental results. The deviation between numerical and experimental tensile strength was within acceptable limits (<5%), confirming model accuracy. Relevant discussion and correlation analysis have been added in Sections 4.4 and 5.

**Comment 13:** All formulations need references.

**Response:** We thank the editor for this insightful comment. We have checked and corrected accordingly.

**Comment 14:** What is the standard deviation in Table 3? For all data? It has no meaning and all data points must have each standard deviation to show the repeatability of testing.

**Response:** Thank you for the observation. The previously reported standard deviation represented the overall variation across trials for each infill pattern, not repeatability. We have included this as the Reviewer-2 instructed us to do so during 1^st^ revision.

**Comment 15:** There is no significant change in Figure 7. Moreover, the repeatability of testing must be reported.

**Response:** Thank you for the valuable suggestion. Figure 7 has been revised with improved resolution and clearer differentiation of the three infill patterns; the updated figure replaces the previous version. Regarding repeatability, each experimental condition was tested in triplicate, and the mean with standard deviation (σ) is reported in Table 3, confirming good experimental consistency.

**Comment 16:** The standard deviation must be added to Figures 8 and 9 and 10.

**Response:** Thank you for the valuable suggestion. The standard deviation values have now been incorporated into Figures 7, 8, and 9 as error bars to reflect experimental variability. These values are consistent with those reported in Table 3. The revised figures improve statistical transparency and enhance the reliability of the presented tensile strength comparisons.

**Comment 17:** Why reverse effect for triangular compared to others in Figures 8 and 9?

**Response:** Thank you for asking an important question. The reverse trend observed for the Triangular pattern in Figures 8 and 9 is attributed to geometry–thickness interaction effects. At higher layer thickness and density, stress concentration at acute node junctions and incomplete interlayer bonding reduce load transfer efficiency, unlike Cubic and Zig-zag patterns, which provide more uniform stress distribution.

**Comment 18:** The discussion is poor. The discussion is not just a description. All obtained results must be compared to other results of other articles.

**Response:** Thank you for the valuable suggestion. The Discussion section has been thoroughly revised to incorporate quantitative comparisons with recent studies on Wood-PLA and PLA-based composites (e.g., Morvayová et al., 2024; Mishra et al., 2024–2025). The obtained tensile strength (46.41 MPa) is critically benchmarked against reported ranges, highlighting the relative improvement achieved through the integrated Taguchi–PSO framework.

**Comment 19:** All features must be added to the SEM images.

**Response:** Thank you for the valuable suggestion. All SEM micrographs have been revised to include clearly labelled features with scale bar.

**Comment 20:** The fracture behaviour must be described in details for SEM images, compared to the literature.

**Response:** Thank you for the valuable suggestion. The SEM fractographs now detail failure mechanisms including fiber pull-out, interfacial debonding, micro-void coalescence, and brittle matrix cracking. The cubic pattern exhibits reduced voids and stronger fiber–matrix adhesion, consistent with higher tensile strength. These observations are critically compared with recent Wood-PLA literature, confirming similar fracture morphology trends.

**Comment 21:** In Table 4, p-value must be added. What is the p-value for lack of fit?

**Response:** Thank you for the observation. The p-values have now been calculated and incorporated into Table 4. The p-value for lack of fit (error term) is 0.041, indicating statistical significance at the 95% confidence level. The revised ANOVA table now clearly reports all corresponding p-values.

**Comment 22:** The structure is confusing, the main text must have an introduction, the research method, results and discussion, conclusions, and references.

**Response:** Thank you for the valuable suggestion. The manuscript has been revised to incorporate several recent (2023–2024) and relevant references on PLA/Wood composites and FDM-based biocomposites. These additions have been integrated into the Introduction and Discussion sections to strengthen the contextual background, compare recent findings, and enhance the overall technical depth and relevance of the study.

**Comment 23:** The error analysis must be added for the results in Figures 13-15.

**Response:** Thank you for the valuable suggestion. Error analysis has now been incorporated in Figures 13–15 by including percentage deviation between experimental, FEM, and regression results has been quantified and discussed to clearly demonstrate model accuracy and result reliability.

**Comment 24:** “Conclusion” must be changed to “Conclusions”.

**Response:** Thank you for the valuable suggestion. The manuscript has been revised accordingly.

**Comment 25:** The conclusions part is lengthy. It should be shortened.

**Response:** Thank you for the valuable suggestion. The conclusions has been already revised accordingly.

**Comment 26:** The conclusions part must be rewritten one by one, in bullets to show the novelty.

**Response:** Thank you for the valuable suggestion. The conclusions has been already revised accordingly and rewritten one by one in bullet points.

The conclusions has been already revised once and converted from bullet points to paragraph according to the instruction given by the Reviewer 1&4 during 1^st^ revision.

**Reviewer-1**

**Comment 1:**

Author has answered all the questions raised perfectly. Hence, I recommend acceptance of the manuscript in its current form.

**Response:** We sincerely thank the reviewer for the careful evaluation of our manuscript and for the positive recommendation for acceptance. We appreciate the acknowledgment that all queries and comments have been addressed satisfactorily. We are grateful for the constructive review process, which has helped improve the overall clarity, technical depth, and quality of the manuscript.

**Reviewer-2**

**Comment 1:** The authors have carefully addressed all the reviewers’ comments and have made the suggested revisions accordingly. The manuscript has been significantly improved in terms of clarity, technical quality, and overall presentation. The study is now well-structured, scientifically sound, and clearly presented. Therefore, the article can be published in its present form.

**Response:** We sincerely thank the reviewer for the thorough evaluation of our revised manuscript and for the positive recommendation for publication. We greatly appreciate the acknowledgment that all reviewer comments have been carefully addressed and that the manuscript has improved in clarity, technical depth, and overall presentation.

**Reviewer-4**

**Comment 1:** The manuscript investigates the optimization of tensile strength in FDM-printed PLA-wood composites using a Taguchi–ANOVA–PSO framework supported by finite element analysis. The authors have satisfactorily addressed the reviewers’ previous comments. Overall, the manuscript shows clear improvement and scientific soundness. Only minor points are suggested below to further improve clarity and contextual positioning.

**Response:** We sincerely thank the Reviewer for the positive and encouraging assessment of our revised manuscript. We appreciate the recognition that the manuscript has substantially improved in clarity, technical rigor, and overall scientific soundness.

**Comment 2:** The background could be strengthened by citing recent studies on FDM-printed PLA–wood bio-composites focusing on material behavior and mechanical performance, for example:

[1] https://doi.org/10.1016/j.ijfatigue.2025.108876

[2] https://doi.org/10.1016/j.jmrt.2025.08.025

**Response:** We sincerely thank the reviewer for the valuable suggestion. As recommended, we have strengthened the Introduction section by incorporating recent studies on FDM-printed PLA–wood bio-composites, including the works cited (DOI: 10.1016/j.ijfatigue.2025.108876 and 10.1016/j.jmrt.2025.08.025).

**Comment 3:** In the PSO formulation, parameters such as print speed and build orientation are mentioned, although they are not varied experimentally. The authors are encouraged to clarify that these parameters were kept constant or to revise the formulation for consistency.

**Response:** We thank the reviewer for this important observation. In the present experimental design, only three process parameters (layer thickness, infill density, and nozzle temperature) were systematically varied using the Taguchi L9 orthogonal array. Parameters such as print speed (40 mm/s) and build orientation were intentionally kept constant throughout all experimental trials to eliminate confounding effects and ensure controlled comparison of the selected factors.

The PSO formulation initially presented a generalized parameter vector to illustrate its broader applicability in additive manufacturing optimization. However, in this study, the optimization search space was restricted strictly to the experimentally varied parameters. The manuscript has been revised accordingly to explicitly clarify that print speed and build orientation were treated as fixed control variables and were not included in the optimization domain. This revision ensures methodological consistency between the experimental design and the PSO formulation.

**Comment 4:** The discussion would benefit from a very brief quantitative comparison of the achieved optimal tensile strength with values reported in selected recent PLA-Wood FDM studies, to further highlight the significance of the results.

**Response:** Thank you for this valuable suggestion. A concise quantitative comparison has now been incorporated in Section 4.2.5 (Discussion) to contextualize the optimized tensile strength achieved in the present work. This quantitative benchmarking highlights the effectiveness of the integrated Taguchi–ANOVA–PSO approach in achieving superior mechanical performance relative to conventional statistical optimization methods.

The authors would like give their sincere gratitude to the Editor(s) and the Reviewers for suggesting modifications and pointing out important errors in the manuscript for improving the quality of it.
