# Supplementary material for: Enhancing tensile strength of 3D-printed wood-PLA composites via a particle swarm optimization framework
Source: PLoS One. 2026 Mar 24;21(3):e0344945. doi: 10.1371/journal.pone.0344945 (PMC13012462; doi:10.1371/journal.pone.0344945)
Supplement: Attachment — Submitted filename: Response to Reviewers (R3).docx [file pone.0344945.s005.docx]

**PONE-D-25-58246R2**

**Title:** Enhancing Tensile Strength of 3D-Printed Wood-PLA Composites via a Particle Swarm Optimization Framework

| **Sl. No.** | **Suggestions** | **Responses** | **Location** |
| --- | --- | --- | --- |
| 1 | "SEM", "ANOVA", and "SDG 9 and 12" are not keywords. Remove them. | We sincerely thank the editor for this valuable observation. We agree that “SEM,” “ANOVA,” and “SDG 9 and 12” represent analytical techniques and sustainability alignment indicators rather than core subject descriptors of the study. Accordingly, these terms have been removed from the keyword list. | Keywords |
| 2 | The abbreviations should not use for keywords. | We sincerely thank the editor for this valuable observation. In accordance with the journal guidelines, abbreviations have been removed from the keyword section and replaced with their full forms to improve clarity, indexing accuracy, and discoverability. The revised keywords now present all terms in their expanded form without abbreviations. |  |
| 3 | All abbreviations must be defined at first mentioning, such as SDG, FFF, etc. | We sincerely thank the editor for highlighting the need for clarity in abbreviation usage. All abbreviations, including SDG (Sustainable Development Goals), FFF (Fused Filament Fabrication), FDM (Fused Deposition Modelling), PSO (Particle Swarm Optimization), ANOVA (Analysis of Variance), and FEA (Finite Element Analysis), have now been defined at their first occurrence in the revised manuscript. The manuscript has been carefully reviewed to ensure consistency and completeness in abbreviation expansion throughout the text. | 1. Abstract 2. Introduction |
| 4 | As mentioned before, the introduction is lengthy. It must be shortened into 2 pages. | We sincerely thank the Editor for this constructive observation. We agree that the Introduction section was comparatively extensive in its original form. Accordingly, the Introduction has been carefully revised and condensed to improve clarity, focus, and readability while retaining essential scientific context and key references. | Introduction has been Revised |
| 5 | Using 52 references for an introduction has no meaning. Some of them can be used for the discussion. | We sincerely thank the editor for this valuable observation. We agree that the Introduction section should focus primarily on establishing the research gap and contextual relevance rather than providing an exhaustive literature survey. Accordingly, we have carefully revised the Introduction by consolidating related references, removing redundancy, and transferring several application-oriented and comparative studies to the Discussion section. | 4.2.5 Discussion |
| 6 | The repeatability of testing must be mentioned in the main text. | We sincerely thank the editor for highlighting the importance of experimental repeatability. In the revised manuscript, we have explicitly clarified the repeatability protocol followed during tensile testing. For each parameter combination, three independent specimens were fabricated and tested under identical conditions, and the average tensile strength value was reported. Additionally, the corresponding standard deviation values have been included to demonstrate measurement consistency and experimental reliability. | The repeatability details have now been incorporated in Section 4.1 (Tensile Strength Analysis) of the revised manuscript. |
| 7 | Generally, the structure is confusing. The main text must have an introduction, research method, results and discussion, conclusions, and references. | We sincerely thank the editor for highlighting the structural organization of the manuscript. We carefully revised the entire manuscript to ensure clear alignment with the standard scientific format consisting of: Introduction, Materials & Method, Results & Discussion, Conclusions, and References. | 1. **INTRODUCTION** 2. **MATERIALS & METHOD** 3. **RESULTS & DISCUSSION** 4. **CONCLUSION** |
| 8 | The details of FEA must be moved to the second part, research method. |  |  |
| 9 | Figure 4(b) must be moved to the third part, results and discussion. |  |  |
| 10 | Check all parts to be in a correct section, for the introduction, research method, results and discussion, conclusions, and references. |  |  |
| 11 | The standard deviation must be removed from Table 3. It has no meaning. For all data and their repeatability, the standard deviation must be reported, for each data point. | We sincerely thank the editor for this insightful observation. We agree that reporting a single pooled standard deviation at the bottom of Table 3 does not sufficiently represent experimental repeatability for individual parameter combinations. In the revised manuscript, the global standard deviation row has been removed. Instead, standard deviation values corresponding to the three repeated tensile tests have now been calculated and reported for each individual experimental trial. This modification provides a clearer representation of measurement dispersion and experimental reliability for every parameter set. The revised Table 3 now includes mean ± standard deviation values for each infill pattern, thereby improving statistical transparency and reproducibility of the results. | Revised Table 3 |
| 12 | Figure 6 does not show the repeatability of testing. | We sincerely thank the editor for this valuable observation. Repeatability and statistical reliability are critical in mechanical characterization studies. In the revised manuscript, we have clarified that each tensile configuration was tested with three independent specimens under identical printing and testing conditions. The mean stress–strain curve is now reported in Figure 6, and the associated standard deviation values have been explicitly stated in the text. Additionally, the statistical consistency is supported through ANOVA and low standard deviation values (σ = 2.99–3.57 MPa), confirming good experimental repeatability. The revised manuscript now clearly reflects this information. | - After first paragraph (after Table 3 reference) - Replace existing caption (Fig 6). - After first paragraph explaining stress–strain curve |
| 13 | No references were added for formulations. | We sincerely thank the editor for highlighting this important point. Appropriate references have now been incorporated to support all mathematical formulations presented in the manuscript, including the Particle Swarm Optimization (PSO) velocity and position update equations, as well as the objective function formulation. These references are now cited in accordance with the journal format using references [10–15] from the existing reference list. The revised manuscript clearly attributes the standard PSO equations and optimization framework to established literature while retaining the original experimental implementation specific to this study. | **Section 3.3**   - At end of objective function explanation - First paragraph of PSO section - Immediately below velocity equation |
| 14 | No details were added to the main text for Comment 17 about the reason for the obtained results (reverse effect for triangular). | We sincerely thank the Editor for this valuable observation. We agree that the reverse tensile strength trend observed in the triangular infill configuration required a clearer mechanistic explanation in the main text. Accordingly, we have expanded Section 4.2.4 to provide a detailed structural and stress-transfer interpretation supported by interlayer bonding behavior, geometric load redistribution characteristics, and SEM-based fracture evidence. The revised discussion now clarifies why the triangular pattern exhibits reduced tensile performance at higher layer thickness despite its geometric stability. The added explanation strengthens the scientific interpretation of the observed anomaly. | New paragraph added in 3.2 heading. |
| 15 | Still, the discussion is poor. Only one paragraph is added which is so simple and not enough. The authors must use more time to address all comments very carefully. | We sincerely thank the editor for this valuable observation. We fully agree that the earlier version of the manuscript did not provide sufficient depth in the discussion section. In the revised manuscript, the discussion has been substantially expanded and technically strengthened. | Added a new session  **3.1** Mechanistic Interpretation and Literature Correlation |
| 16 | 1. No features were added to the SEM images. | We sincerely thank the editor for this valuable observation. In the original submission, the SEM images were presented without graphical annotations, which may have limited clarity in identifying fracture features. In the revised manuscript, we have updated all SEM micrographs (Figure 10) by adding clear graphical markers and labels highlighting key fracture features | Marked in figure 10 (a, b, c) |
|  | 2. Moreover, the fracture behaviors must be compared to the literature and described which marks were seen and which fracture behaviors were seen, ductile or brittle, why, etc. | We sincerely thank the editor for highlighting the need for deeper fracture behavior interpretation. In the revised manuscript, the SEM fractographic analysis has been significantly expanded to (i) classify the fracture mode (brittle vs. ductile), (ii) identify characteristic fracture features (fiber pull-out, matrix cracking, interfacial debonding, void coalescence), and (iii) compare the observed mechanisms with recent Wood-PLA and PLA-based FDM composite studies [7], [9], [45], [51], [52]. | Added new paragraph as: 3.2.1 Fracture Morphology and Failure Mechanism Analysis |
| 17 | In Table 4, the p-value is high for each parameter and therefore, the regression has no meaning and they have no effect on the objective. The details must be described in the main text. This is why I mentioned the used method is not good. Use another method of regression. | We sincerely thank the editor for the valuable observation regarding the statistical significance of the regression model. We agree that the originally reported p-values in Table 4 require clearer interpretation and additional statistical validation. The relatively high p-values are primarily attributed to the limited experimental degrees of freedom inherent to the Taguchi L9 orthogonal array design (df = 2 for error), which restricts statistical power. | Added a new content in 3.5 Heading, Also one more new subsection “3.5.1 Model Adequacy and Regression Validation” has been added for your kind approval. |
| 18 | The p-value of lack of fit must be reported. It is low and therefore; it shows a not proper regression. | We thank the editor for this important observation. We agree that reporting the lack-of-fit (LoF) test is necessary to evaluate regression adequacy. In the revised manuscript, the LoF p-value has now been explicitly reported and interpreted in the statistical discussion section. The initial regression model was a first-order (linear) formulation. A statistically significant lack-of-fit (low p-value) indicates that curvature and/or interaction effects are present in the response behavior and are not fully captured by a linear model. In FDM-based composite systems, nonlinear interactions between layer thickness, infill density, and nozzle temperature are physically expected due to interlayer diffusion, void evolution, and stress transfer mechanisms. To address this, we have replaced the linear regression model with a second-order response surface regression (RSM) model incorporating quadratic and interaction terms. The revised model resolves the lack-of-fit concern and significantly improves predictive capability. The manuscript has been updated accordingly. | - Immediately after Table 4 - Replace current Table 5 (linear equations) - Add as a short paragraph “3.5.2 Model Adequacy and Regression Validation” |
