# Supplementary material for: Enhancing tensile strength of 3D-printed wood-PLA composites via a particle swarm optimization framework
Source: PLoS One. 2026 Mar 24;21(3):e0344945. doi: 10.1371/journal.pone.0344945 (PMC13012462; doi:10.1371/journal.pone.0344945)
Supplement: Attachment — Submitted filename: Response to Reviewers (R4).docx [file pone.0344945.s006.docx]

**PONE-D-25-58246R3**

**Title:** Enhancing Tensile Strength of 3D-Printed Wood-PLA Composites via a Particle Swarm Optimization Framework

| **Sl. No.** | **Suggestions** | **Responses** | **Location** |
| --- | --- | --- | --- |
| 1 | It has no meaning to write "+/-SD" in Table 3. The value of SD must be mentioned! | Thank you for your valuable observation. We agree that indicating only “±SD” without numerical values is incomplete. Accordingly, Table 3 has been revised to include the actual standard deviation (SD) values along with the mean results for each infill configuration. This modification provides clearer quantitative information on data variability and improves the statistical transparency and reliability of the reported results. | 3.RESULTS & DISCUSSION |
| 2 | Still, the authors have 52 references in the introduction! Not meaningful. | We sincerely thank the editor for this valuable observation. We have revised the Introduction part accordingly and reduced it to 40 references. | 1.INTRODUCTION |
| 3 | All all data to Figure 6 to show the repeatability of testing. | Thank you for this valuable suggestion. Figure 6 has been revised to include all individual stress–strain curves obtained from three repeated tensile tests for each infill configuration, along with their corresponding average curve. The updated figure clearly demonstrates the excellent repeatability and consistency of the experimental results, with minimal deviation among repeated tests. The revised figure and caption have been updated in the manuscript. | 3.RESULTS & DISCUSSION |
